# Management of Trigeminal Neuralgia with Botulinum Toxin Type A: Report of Two Cases

**DOI:** 10.3390/dj10110207

**Published:** 2022-11-03

**Authors:** Craig Pearl, Brendan Moxley, Andrew Perry, Nagi Demian, Cyndie Dallaire-Giroux

**Affiliations:** 1 Department of Oral and Maxillofacial Surgery, The University of Texas Health Science Center at Houston, Houston, TX 77054, USA; 2 School of Dentistry, The University of Texas Health Science Center at Houston, Houston, TX 77054, USA

**Keywords:** trigeminal neuralgia, botulinum toxin type A, inferior alveolar nerve, multiple sclerosis, pain relief, orofacial pain, trigeminal neuropathy

## Abstract

Trigeminal neuralgia is a chronic pain condition associated with sharp, shock-like pain in one or more divisions of the trigeminal nerve. For patients who do not respond well to pharmacotherapy, there is growing evidence that Botulinum toxin type A injections into the trigeminal ganglion provide pain relief for several weeks up to several months at a time. One option is to administer injections into the trigeminal ganglion in Meckel’s cave by inserting a needle through the Pterygopalatine Fossa using fluoroscopy to guide and confirm the proper needle placement. However, there is evidence that Botulinum toxin travels across nerve synapses; thus, injecting directly into the trigeminal ganglion may not be necessary. We present two patients with a confirmed diagnosis of trigeminal neuralgia who were treated by injecting Botulinum toxin type A intraorally into the mental foramen which resulted in 6 months or longer of pain relief. Injections into the mental foramen are much easier to administer than those administered directly into the trigeminal ganglion, and both patients treated with this technique experienced comparable results to what can be expected from traditional fluoroscopy-guided botulinum toxin injections. Though more research is needed, these cases potentially imply that a less-invasive injection may be sufficient in managing trigeminal neuralgia-related pain.

## 1. Introduction

Trigeminal neuralgia (TN) is a chronic neurological disorder characterized by sharp, shock-like pain in one or two of the three main divisions of the trigeminal nerve: namely, the frontal (V1), maxillary (V2), and mandibular (V3) nerves [1]. It affects 4–28.9/100,000 people, typically presenting in females over 50 years of age because of intracranial neurovascular compression, although less frequently, it may occur in younger patients because of chronic illnesses such as multiple sclerosis [2]. Frequently, the pain begins in one branch and over a period may involve more than one branch of the trigeminal nerve [3]. Trigeminal neuralgia may present earlier in patients with multiple sclerosis (MS); in one study, approximately 15% of patients diagnosed with MS were first diagnosed with TN [4,5].

The first line of treatment includes pharmacotherapy, usually with anticonvulsants and antispasmodic agents, although this management is frequently associated with side effects and variable success [6]. Other interventions may include microvascular decompression, gamma knife radiosurgery, or rhizotomies, each of which carries significant risks and potential adverse effects [7]. Multiple studies show that Botulinum toxin type A (BTX-A) injections into the trigeminal ganglion are effective for trigeminal neuralgia patients. These injections function by cleaving and inactivating SNARE proteins which are responsible for neurotransmitter release within the nerve endings [8]. The reduced neurotransmitter release reduces neuropathic pain in the orofacial region, although the exact mechanism of action of transport is not clear [9]. However, administering these injections often requires radiographic imaging with resultant cumulative radiation exposure and inconvenience due to limited access points to the trigeminal ganglia [10,11,12]. Furthermore, pain relief is temporary, typically lasting anywhere from 6 weeks to 6 months, meaning patients need to return for continual injections [13]. The pterygopalatine fossa can be accessed via a trans-nasal, trans-oral, or more commonly via an infra-zygomatic approach using an ultrasound, computed tomography, or fluoroscopic imaging technique [14,15,16,17,18]. Literature exists on performing these injections using a CAD/CAM printed guide [19] or less commonly without any guide [13]. Regardless, these injections are administered under sedation, and the pterygopalatine fossa is a much less accessible injection site than the peripheral foramina. Injections are frequently performed into the mental nerve for a variety of procedures, most notably, mental nerve blocking with local anesthetic for a variety of dental treatments. If comparable results and pain relief are obtained after injecting BTX-A into one of the peripheral foramina such as the mental foramen, then this minimally invasive technique would likely be favored for trigeminal neuralgia patients.

A literature review showed a paucity of studies testing the efficacy of injecting BTX-A into a peripheral foramen rather than the trigeminal ganglion itself to treat trigeminal neuralgia. Botulinum toxin is known to travel along the peripheral nervous system to the trigeminal ganglion, mediated by axonal organelles that travel via microtubules [20]. In one case report reviewed, BTX-A was injected into the terminal ends of the V1 and V2 branches of the trigeminal nerve to treat intractable trigeminal neuralgia [21]. The patient treated with this technique also noticed a reduction in pain in the V3 (mandibular division), though that site was not injected. This suggests that nerve travel of BTX-A occurs bi-directionally. Notably, the patient did not experience any systemic or adverse effects from the injections.

A PubMed search conducted on 7 July 2022 using MeSH terms including “Trigeminal Neuralgia”, “Botulinum Toxin Type A”, and “Pain Relief” showed only one article where the terminal end of the V3 branch was injected via the mental nerve to manage trigeminal neuralgia. Although the researchers described this study as quasi-experimental, they observed a statistically significant decrease in VAS and frequency of attacks, with 19 of 20 patients experiencing significant pain relief and only 1 patient experiencing minor inferior labial weakness which improved after 2 months [22]. However, one point of weakness for this article is that it does not demonstrate the criteria used to diagnose these patients with trigeminal neuralgia. Given the high misdiagnosis rate [23], it is prudent to adopt a confirmation process to ensure that the patients tested are suffering from the same condition. This case report presents 2 patients whose neurologists determined that they presented clinical features consistent with trigeminal neuralgia. While these two patients were suffering from the same condition, each had a different etiology, with the first patient’s condition being caused by neurovascular compression and the second patient’s condition stemming from MS. Adding to the complexity of the second case was the fact that she suffered inferior alveolar nerve (IAN) damage following the extraction of a deeply impacted lower left wisdom tooth. In each patient, 20 units of BTX-A were injected into the mental foramen after conservative treatment failed.

## 2. Materials and Methods

The injection site is identified initially by assessing the exact location of the mental foramen on a cone-beam computed tomography (CBCT) or panoramic radiograph. Most frequently, the foramen is situated between the apices of the first and second lower premolars or just below the apex of the lower second premolar.

The patients are then assessed intraorally and the correlation with the radiograph is determined. The BTX-A syringe is then prepared using the dilution of 1 cc of non-preserved sterile saline into 50 units of Botulinum toxin A, which is then drawn up into a 1 cc tuberculin syringe with a 27–30-gauge needle. Each 0.1 cc of solution represents 5 units of BTX-A; hence the patient will receive 0.4 cc (20U).

**Figure 1:**
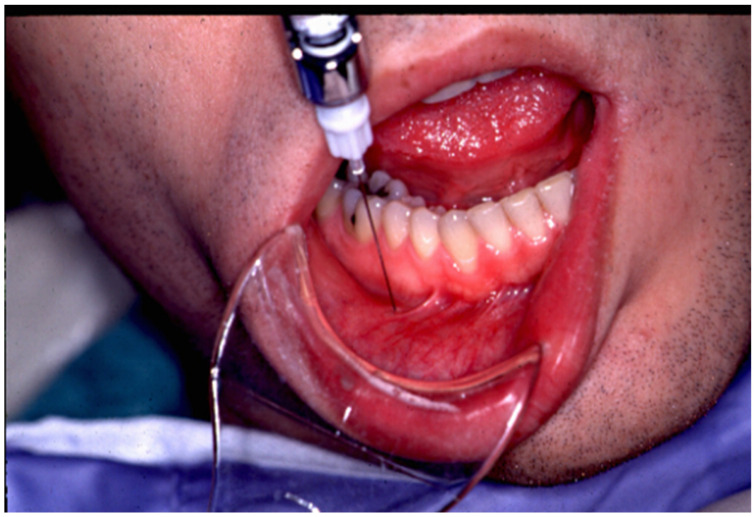
The technique demonstrated is used to locate the mental foramen and deposit a small amount of local anesthetic followed by Botulinum toxin type A as a treatment for trigeminal neuralgia.

The lower lip on the injection side is retracted gently and the foramen is then identified using a dental syringe and needle or directly with the tuberculin syringe and 30 gauge needle. Lidocaine 1% plain may be injected prior to the BTX-A, depending on the patient’s preference. The 0.4 cc of BTX-A is injected at a 45–60-degree angle to the mandible, and the needle is initially advanced 2–3 mm, at which point, it usually contacts the bone of the mandible. Once the foramen is located, the needle can be further advanced an additional 2 mm just beyond the opening of the mental foramen. The BTX-A is then injected slowly to avoid pain or potential nerve injury (see Figure 1). Even without prior local anesthetic, the patient will report paresthesia for a couple of hours, which is frequently welcomed by the patient.

Important to note is the potential confounding variable of using both lidocaine and BTX-A in assessing the efficacy of BTX-A for the management and resolution of trigeminal neuralgia-related pain. In both cases, a diagnostic block using lidocaine plain was performed in the initial consultation to assess if, by eliminating the mandibular division of the trigeminal nerve (V3) as a source, the pain improved only while numb, and as soon as the lidocaine plain wore off 30–60 min later, the pain returned in the exact same intensity and frequency. Performing nerve blocks is crucial in the diagnosis of traumatic trigeminal neuralgia especially [24]. Because this is exactly what was observed in both patients, the longer-term pain relief is more likely attributed to the effects of the Botulinum toxin. However, this is an area of potential further study.

### 2.1. Case 1

Patient 1 is an 82-year-old female who presented to our clinic for a consultation regarding trigeminal neuralgia which was diagnosed by her neurologist in 2019 based on her clinical signs and symptoms as well as a computed tomography angiography (CTA) of the brain confirming the superior cerebellar artery pulsating over the trigeminal root entry zone. The patient reports that the pain is intermittent in nature, but she rates it as a 10/10 on the visual analog scale (VAS) and describes the pain as “a strike of lightning” in her lower left jaw, beginning at the canine area and extending posteriorly. The patient has a 16-year history and cannot recall any inciting events or trauma to the area. She did have multiple dental extractions in the lower left mandible after the symptoms began because of a misdiagnosis on the part of the dentist. Her primary care physician prescribed 300 mg of gabapentin per day, which provided minor relief but gave her a systemic rash, so she was then referred to a neurologist who prescribed 300 mg of carbamazepine per day, which provided only moderate relief. She is unable to tolerate higher dosages of carbamazepine due to the increased severity of the side effects she experiences, namely, severe malaise, lethargy, and confusion. She reports feeling pain in the region of the inferior alveolar nerve as well as in the lingual division, with the lingual branch presenting with a tingling sensation with no alteration in taste sensation.

Due to the classic symptoms described by the patient as well as the positive CTA findings, we agreed with her diagnosis of trigeminal neuralgia. She was made aware of the various management options including continuing with carbamazepine, cryotherapy, neurovascular decompression, gamma knife therapy, trigeminal ganglion nerve blocks, or BTX-A injections. She elected to return to her neurologist to discuss the side effects of the medication and persistent chronic pain.

Nine months later, the patient returned, requesting BTX-A injections as her neurologist was unable to find an optimal dosage for her with pharmacotherapy treatment to minimize pain without resulting in increased side effects. The patient was informed that the use of BTX-A is not an FDA-approved procedure but that there is a growing body of literature supporting BTX-A for the management of TN. The patient was also informed that the injections with local anesthetic usually are placed into the trigeminal ganglion under fluoroscopy; however, we would be injecting BTX-A into the mental foramen on the left and assessing the response. After obtaining informed consent and reviewing the most recent cone beam computed tomography scan to determine the precise location of the mental foramen, one cc of 2% lidocaine plain was administered into the left mental foramen. Once it was confirmed that the patient was anesthetized, twenty units of BTX-A were then administered to the mental foramen. The patient endorsed diminished pain with numbness to the appropriate nerve distribution. This treatment provided 6 weeks of complete pain relief, and at approximately 7 weeks, the patient reported the return of a shock-like pain in the V3 distribution, though with lower intensity and less frequency. It was decided to repeat the BTX-A injections, and following this round of injections, the patient experienced 10 weeks of pain relief. The third treatment provided 5 months of pain relief, and after the fourth treatment, it has been 18 months since the patient received her last BTX-A injection, and she is currently pain-free with no intermittent attacks and is currently not on any medication for TN.

### 2.2. Case 2

Patient 2 is a 34-year-old female who presents for a second opinion for neuralgia-type pain on the left following the surgical removal of an impacted wisdom tooth. Noteworthy diagnoses in the patient’s medical history include multiple sclerosis with slight motor weakness of the lower limbs which is more pronounced on the left side and optic neuritis on the right side, which is what led to the diagnosis of MS by her neurologist.

She developed a slight pericoronitis on her lower wisdom teeth, which coincided with her starting treatment for her MS. On preoperative x-ray, it was determined that the lower left wisdom tooth was vertically impacted, and the apices of the wisdom tooth were noted to be intimately associated with the IAN. At the time of the extraction, the oral surgeon packed the lower left extraction socket with bovine bone xenograft. The patient contacted her surgeon 48 h postoperatively, informing them that she had persistent numbness on the lower left lip and chin. The surgeon reassured her that the tooth was very deep and that this was to be expected. At her one-week follow-up, the patient was informed that the sockets were healing well, and despite the V3 on the left being anesthetic, she was told the sensation would return after a 3–6-week period.

She continued with the surgeon over the subsequent 3-month period, and despite the patient being numb, she reported the onset of significant shock-like pain emanating from the center of the anesthetic side, but the surgeon once again reassured her. They informed her that it was related to her MS, resulting in unpredictable recovery.

**Figure 2:**
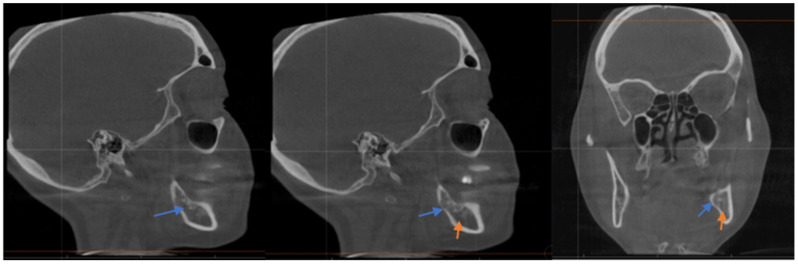
Sagittal CT scan: Note the proximity of the mineralized xenograft in tooth #17 (Blue Arrow) following surgical removal and bone graft procedure to inferior alveolar nerve canal (Orange Arrow).

She sought a second opinion 4 months post-surgery, and she was found to have anesthesia on the left distribution of IAN with intense shock-like pain, consistent with anesthesia dolorosa because of probable IAN damage. The CBCT showed that the xenograft was packed into the base of the socket, which was in direct contact with the IAN, and thus mineralized bovine bone was found to be completely compressing the nerve in the wisdom tooth area (see Figure 2). We discussed the distinct options for management ranging from pharmacological management to surgical intervention; however, she was already on medication for her MS, and the surgical option would involve a sagittal split osteotomy on the left side to try to free the nerve with the possibility of permanent numbness without any guarantee of success, so she was not in favor of the surgery. The patient described her pain stemming from the center of her numb lower left lip and chin region as intense and episodic, though the sensation to her tongue had returned. The patient also reported that the shock-like pain had recently spread over the left maxillary area as well.

We administered an IAN block with 2% lidocaine plain and observed the reduction in the pain proximal to the injection site as well as her V2 pain; however, the intense shock-like pain distal to the injection site in the lower lip on that side remained. Once full sensation had returned, we then gave her a mental nerve block on that side with lidocaine and this resulted in immediate cessation of pain in both V2 and V3, which suggested a diagnosis of a peripheral mediated pain, though extension into V2 was inconsistent with this. We then proceeded to inject Marcaine with Kenalog into the mental nerve on the left to determine if this would provide any longer-term relief.

The patient returned for a 2-week follow-up where she explained that she only had pain relief for as long as the Marcaine was effective. We discussed consulting with her neurologist to change her pregabalin to gabapentin, but she mentioned that the gabapentin made her very light-headed and lethargic. We then discussed that BTX-A has shown positive results when used in managing chronic neuropathic pain. We requested a clearance from her neurologist and discussed her diagnosis since she is presenting with signs and symptoms of both neuropathic pain due to nerve injury as well as TN.

The neurologist saw the patient and they diagnosed her with TN of the V3 and V2 as well as iatrogenic trigeminal neuropathic pain in the V3. They agreed with the BTX-A injections and wished to change her pregabalin to Tegretol, although the patient did not agree to change at that stage. The patient was informed by our service that BTX-A has shown benefits in managing TN, although most of the literature has demonstrated efficacy when injected into the trigeminal ganglion and not into a peripheral branch. When performing the procedure, the mental foramen was located using available radiographs and a dental syringe, and she was given lidocaine plain followed by 20 units of BTX-A into the mental nerve. The patient tolerated the procedure well. At her two-week follow-up appointment, the patient reported that it took 1 week after the procedure for her pain to diminish and subsequently cease. She continued taking her pregabalin during this time.

This pain relief lasted for 5 months, at which point, the patient returned for the exact same regimen of 20 units of BTX-A injection into the left mental foramen. At her 2-week follow-up, the patient indicated that she responded better to the injections this time and that all her pain had gone. She also informed us that her MS had deteriorated significantly, and as a result, her neurologist was starting a whole new management protocol.

The patient was seen at 8 weeks after her second treatment with BTX-A and she remained pain-free, although she also remained anesthetic on the left side with no improvement. She has had this management for 18 months now and has definite benefits; however, her MS has progressively deteriorated which, according to her neurologist, has exacerbated her TN flare-ups.

## 3. Results

Both patients reported a similar decrease in the frequency and intensity of shock-like pain attacks from a mean of 16 per day pre-treatment to no more than 4 post-treatment. The intensity of the pain also significantly decreased from a mean of 10 on the VAS pretreatment to between 2 and 3 post-treatment. This was followed by a period of complete cessation of pain, with patient 2 requiring two weeks as opposed to one week for patient 1.

Irrespective of etiology, the period of pain relief seemed to increase after each consecutive treatment. At the most recent follow-up appointment, both patients reported an improvement in pain and quality of life, which they indicated was significantly better than any other treatment they had previously received. The patients stopped their prescribed TN medication under the supervision of their neurologist after receiving this treatment.

This case report is organized with both cases together because, despite the patients presenting very different etiologies of their trigeminal neuralgia-like pain, the BTX-A toxin appeared to be effective in offering pain relief for both.

When comparing the response to treatment between the patients, the patient with neurovascular compression-related TN responded quicker after each treatment and seemed to experience a far higher quality of life and overall more consistent pain relief. The patient with MS-related TN and iatrogenic neuropathic pain also required two treatments with BTX-A before reducing and stopping medication for pain as opposed to patient 1 who reduced and stopped medication after one BTX-A treatment.

Significantly, none of the patients reported any prolonged alteration in the mental nerve distribution following injecting BTX-A into the mental foramen. Patient 1 reported paresthesia for 48 h, while patient 2 reported no change as they were already anesthetic from the initial wisdom tooth extraction and bone graft.

These results are consistent with what has been reported in the literature for traditional BTX-A injections into the trigeminal ganglion; however, these patients were treated with an easier, less-invasive technique and were spared from additional radiation exposure to achieve a similar outcome.

## 4. Discussion

The method of action of BTX-A traveling along the nerve is not entirely understood, though recent animal models provide new insights. These studies reveal that the BTX-A can travel in either retrograde [25] or anterograde transport in the nerve axons due to transport on the microtubules [26]. BTX-A can also travel across nerve synapses to adjacent neurons [27] and glial cells [28].

It is also uncertain how BTX-A acts to reduce pain when injected into a nerve ending. Regardless of the mechanism of action, the analgesic effects of BTX-A are well-documented [29,30]. A popular explanation for the analgesic effects of BTX-A includes that it acts on sensitized nociceptive fibers by blocking the protein kinase C potentiation of transient receptor potential vanilloid 1 (TRPV1), which is a capsaicin and heat-sensitive ion channel expressed in nociceptors [30]. As trigeminal neuralgia pain is described as feeling like an electric shock, it is fitting that BTX-A injections into the nerve would provide analgesic relief for affected patients if this were the method of action. Interestingly, in the second patient’s case, the BTX-A that was injected into the mental nerve appeared to provide significant improvement for her TN-related pain as well as her neuropathic pain associated with the IAN injury.

In the second case, it is important to emphasize that the patient manifested symptoms of TN and MS prior to the IAN damage; however, after the nerve damage was sustained, she experienced more intense TN-like pain with the added numbness from the injury. Thus, it appears that the nerve damage exacerbated her TN.

What was noted when comparing the efficacy of this treatment between the two patients was that, despite the effectiveness of this treatment for both patients, the patient with MS and iatrogenic nerve injury was far more variable in her response. There also seemed to be a correlation between the patient’s MS flare-ups and alterations in her MS medication, which may pose a confounding variable in determining their response to BTX-A. Despite the variability in patient 2′s case, it may be that this treatment is effective not only for trigeminal neuralgia but also for trigeminal neuropathy, although far more research is needed in this area.

## 5. Conclusions

While double-blind studies are needed to suggest the efficacy of this technique, these two patients report effective pain relief. The current literature and our experience, though both limited, indicate that this treatment modality is safe and effective with no local or systemic adverse effects. For these two patients, the regimen described in this report is fast-acting and long-lasting; however, this is an evolving technique, and additional research is necessary to identify the optimal dosage, the timing of each injection, and its effectiveness when compared to existing treatments for different causes of TN.

## Data Availability

Not applicable.

## Abbreviations

TN	Trigeminal Neuralgia
MS	Multiple Sclerosis
BTX-A	Botulinum Toxin Type A
MRI	Magnetic Resonance Imaging
CBCT	Cone-beam Computed Tomography
CAD/CAM	Computer-Aided Design/Computer-Aided Manufacturing
IAN	Inferior Alveolar Nerve
CTA	Computed Tomography Angiography
VAS	Visual Analog Scale
FDA	Food and Drug Administration
TRPV1	Transient Receptor Potential Vanilloid 1
